# Signet ring cell carcinoma of the ampulla of Vater: Immunophenotype and differentiation

**DOI:** 10.3892/ol.2014.2344

**Published:** 2014-07-11

**Authors:** XUE WEN, WEIQIANG WU, BO WANG, HONGTIAN YAO, XIAODONG TENG

**Affiliations:** Department of Pathology, The First Affiliated Hospital, College of Medicine, Zhejiang University, Hangzhou, Zhejiang 310003, P.R. China

**Keywords:** ampulla of Vater, signet ring cell carcinoma, immunophenotype, differentiation

## Abstract

Signet ring cell carcinoma (SRC) of the ampulla of Vater is extremely rare and the histogenesis remains unknown. In the present study, to investigate the immunohistochemical phenotypes, discuss the histological origin and evaluate the correlation between the immunohistochemical phenotypes and survival of ampullary SRC patients, a retrospective review was conducted. This included all ampullary carcinoma patients treated at The First Affiliated Hospital, College of Medicine, Zhejiang University, and was performed over a five-year period between 2008 and 2012. Eight resected ampullary SRC specimens were examined histopathologically and immunohistochemically, using cytokeratin (CK) and mucin (MUC) immunohistochemical phenotypes. Of all 162 patients with ampullary lesions, eight cases (4.9%) of ampullary SRC were identified. Immunohistochemical analyses of the eight cases revealed the positive expression of CK7 in five, CK19 in seven, CK20 in one, MUC1 in five, MUC2 in three, caudal-related homeobox transcription factor 2 in one, MUC5AC in seven and MUC6 in four of the eight cases, while loss of E-cadherin and β-catenin was observed in four of the eight cases. According to immunohistochemical classification, ampullary SRC can be classified into four subtypes: Intestinal (I), pancreatobiliary (PB), gastric and mixed types (composed of I mucosa lining and PB epithelium). Patients with the I-type ampullary SRC demonstrated a more favorable prognosis than that of patients with the PB-type ampullary SRC. Additionally, patients with ampullary SRC of I or PB type with gastric differentiation may have a worse prognosis than others. The coexpression of the E-cadherin/β-catenin complex may also indicate poor prognosis in PB-type ampullary SRC. In conclusion, the clinical five-year follow-up of the patients with pure SRC was more positive than that of those with I-, PB-, gastric- or mixed-type ampullary SRC. The coexpression of the E-cadherin/β-catenin complex may present a poor prognosis in the PB type of ampullary SRC.

## Introduction

Ampullary carcinoma is a rare entity accounting for only 0.2% of all gastrointestinal malignancies and <6% of all periampullary cancers (1,2). Signet ring cell carcinoma (SRC) is extremely uncommon in the ampulla of Vater; few cases have been previously described and only mini-reviews are available (3–11). In the current study, owing to its location at the ampulla, obstructive jaundice was the most common symptom. The majority of patients also exhibited dilation of the common bilary duct and the main pancreatic duct, as well as an enhanced mass lesion in the ampulla of Vater, as determined by helical computed tomography (CT). At present, surgical resection is performed as the only curative treatment, with pancreatoduodenectomy or ampullectomy the most common options. As for prognosis, Hara *et al* (12) showed that SRC localized in the ampulla of Vater has a poor prognosis and lymph node involvement is also regarded as a key prognostic factor. Certain authors have also demonstrated that initial surgical resection with adjuvant therapy may not provide a survival benefit in patients without lymph node invasion (5). Due to its marked association with gastric mucosa, Blundell *et al* (13) hypothesized the origin of SRC only in the presence of gastric mucosa/metaplasia. Owing to SRCs expression of the caudal-related homeobox transcription factor 2 (CDX2) and mucin (MUC) 2, certain authors have also suggested that they are considered to be variants of intestinal (I)-type adenocarcinoma (14). Gheza *et al* (15) reported an additional histogenesis of SRC differentiation from the pancreatobiliary (PB) type of adenocarcinoma. In the present study, to discuss the histological origin and explore the correlation between the immunohistochemical phenotypes and survival of ampullary SRC, the establishment of a simple ampullary SRC classification system was attempted based on the immunohistochemical staining of cytokeratin (CK) and MUC in eight cases.

## Patients and methods

### Patients

A retrospective review of the records of all the patients diagnosed with ampullary cancer at the First Affiliated Hospital, (Hangzhou, China) was performed between January 2008 and December 2012. The study was approved by the ethics committee of The First Affiliated Hospital, College of Medicine, Zhejiang University (Hangzhou, China) and written informed consent was obtained for each patient. A total of 162 patients were identified with ampullary neoplasms and their final histopathological diagnosis was determined from pancreaticoduodenectomy specimens. Of these, 152 (93.8%) were adenocarcinoma, eight (4.9%) were SRC and two (1.2%) were neuroendocrine tumors. Of the patients with SRC, four were male and four were female with an average age of 60 years (range, 42–75 years). The patients’ clinical details, surgical outcome with five-year follow-up and treatment were reviewed. All patients underwent pancreaticoduodenectomy and one patient underwent extended lymphadenectomy.

### Antibodies

The signet ring cells were stained immunohistochemically using the following antibodies: Monoclonal mouse anti-human CK7 (clone OV-TL 12/30; 1:200; Dako Agilent Technologies Company, Copenhagen, Denmark), CK20 (clone Ks20.8; 1:80; Dako Agilent Technologies Company), CDX2 (clone DAK-CDX2; 1:200; Dako Agilent Technologies Company), MUC1 (clone MRQ-17; 1:80; Cell Marque Corporation, Rocklin, CA, USA), MUC2 (clone CCP58; 1:100; Beijing Zhongshan Golden Bridge Biotechnology Co., Ltd., Beijing, China), MUC5AC (clone MRQ-19; 1:100; Beijing Zhongshan Golden Bridge Biotechnology Co., Ltd.), MUC6 (clone MRQ-20; 1:100; Beijing Zhongshan Golden Bridge Biotechnology Co., Ltd.), E-cadherin (clone NCH-38; 1:100; Dako Agilent Technologies Company), β-catenin (clone β-catenin-1; 1:200; Dako Agilent Technologies Company) and CD10 (clone 56C6; 1:50; Dako Agilent Technologies Company).

### Immunohistochemisty

After being fixed in 10% natural-buffered formalin for ~24 h, all tissue specimens were embedded in paraffin, sectioned (4-μm thick), and then stained with hematoxylin and eosin. The histopathological variables included lymphovascular and tumor size, and all tumors were staged according to the WHO classification for ampullary carcinoma (16); cases in which signet ring cells constituted >50% of the adenocarcinoma were regarded as SRC. The histology of all tumors was examined by experienced pathologists with no prior knowledge of the clinical or pathological outcomes. Positive and negative controls were produced for all of the antibodies tested. The staining score was evaluated as the percentage ratio of stained signet ring cells to the total number of cells evaluated. The signet ring cells showing <10% tumor cell positivity were regarded as negative, 10–50% tumor cell positivity were designated as focal positive and >50% tumor cell positivity were considered diffuse positive.

## Results

### Clinical and pathological features

The pertinent clinical features are summarized in the Tables I and II. Of all 162 patients with ampullary lesions, eight cases (4.9%) were diagnosed as SRC, with a mean age at presentation of 60 years. The presenting symptoms information was available for all eight cases. Five patients presented with jaundice, weight loss and abdominal pain associated with the tumor, while two patients had fever and vomiting. The remaining case was asymptomatic and the disease was recognized by routine abdominal ultrasound and CT with diffuse ampulla of Vater wall thickening. The predominant radiological observations were long segmental wall thickening, dilatation of the common biliary tract and the main pancreatic duct, and an enhanced mass lesion. All patients underwent surgical pancreaticoduodenectomy and one patient underwent extended lymphadenectomy. The overall tumor size ranged between 1.2 and 9.5 cm, and the mean size was 3.8 cm. Postoperative adjuvant chemotherapy was performed in four cases and one patient received radiotherapy. Follow-up data were available for all eight patients; five patients are alive (cases 1, 2, 6, 7 and 8), including two with clinical evidence of lymph node metastatic disease (cases 1 and 6); one patient succumbed to brain and bone metastases at two years following diagnosis (case 4); one patient succumbed to liver metastases at one and a half years following diagnosis (case 3); and one patient succumbed to lymph node metastases at nine months following diagnosis (case 5).

### Immunohistochemical examination

**Figure 1:**
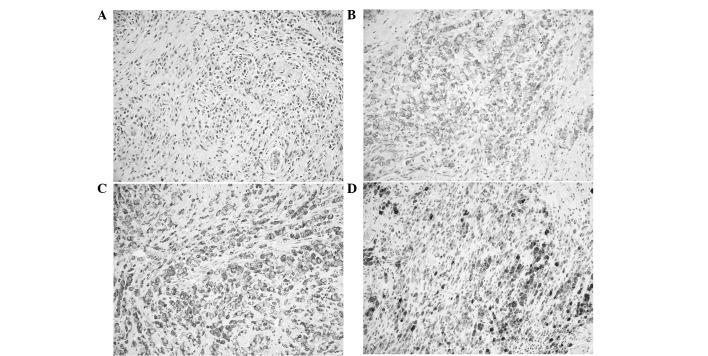
Expression of CK and MUC in PB-type ampullary signet ring cell carcinoma. (A) Hematoxylin and eosin staining of PB-type carcinomas. (B) Diffuse expression of CK7. (C) Strong immunopositive staining for CK19. (D) Positive expression of MUC1 (magnification, ×200). CK, cytokeratin; PB, pancreatobiliary; MUC, mucin.

**Figure 2:**
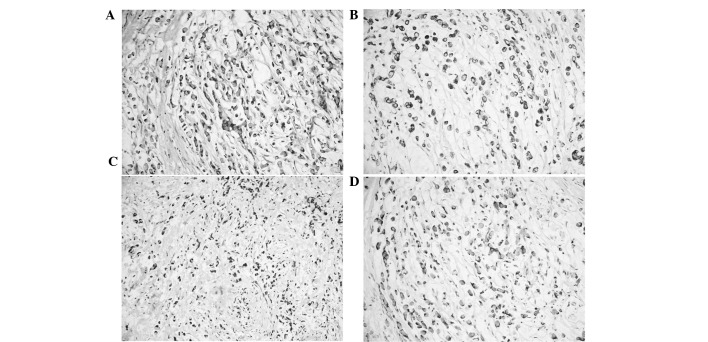
Expression of CK, CDX2 and MUC for I-type ampullary signet ring cell carcinoma. (A) Hematoxylin and eosin staining of I-type carcinomas. (B) Strong immunopositivity for CK20. (C) Positive expression of CDX2. (D) Immunopositivity for MUC2 (magnification, ×200). I, intestinal; CK, cytokeratin; MUC, mucin; CDX2, caudal-related homeobox transcription factor 2.

The immunohistochemical results of the eight cases are shown in Table III. In total, four out of the eight ampullary SRCs showed poorly differentiated adenocarcinoma with prominent signet ring cell features; of these, three showed CK7^+^, CK19^+^ and MUC1^+^ staining (cases 5, 3 and 6; Fig. 1) and one case showed positive immunoreactivity for CD10 (case 3). Immunoreactivity for MUC5AC and MUC6 was evident in three cases (cases 1, 3 and 5). Only one case showed prominent signet ring cells floating in a pool of mucus which were positive for CK20, CDX2 and MUC2 (case 2; Fig. 2). Two pure SRCs were positive for CK7, CK19 and MUC1 (cases 4 and 7), and one pure SRC was positive for CK19, MUC2 and MU5AC (case 8); however, only one case was positive for MUC5AC and MUC6 (case 4). Four cases showed loss of expression of the E-cadherin/β-catenin complex (cases 1, 2, 6 and 8). The comparison between these immunoprofiles and those of the eight cases of ampullary SRC are shown in Table III. In the present study, according to the immunohistochemical results, ampullary SRC may be classified into the following four subtypes: I-, PB-, gastric- and mixed-type (composed of I mucosa lining and PB epithelium). One I-type tumor patient and one of the five PB-type tumor patients, as well as two patients with mixed-type tumors were classified as stage III according to the WHO criteria (16). Two of the PB-type tumor patients were classified as stage IIB, while one gastric-type and two PB-type tumor patients were classified as stage IIA. No lymph node metastasis was documented in two of the five cases of PB-type ampullary SRC.

## Discussion

SRC is a rare tumor that arises in a number of organs, including the stomach, gallbladder, breast and urinary bladder; particularly in the stomach (17). Sekoguchi and Mizumoto (3) first reported this histological pattern in 1979, and individual case reports or small series of a few cases have since been reported in the English language literature (4–11). In accordance with the WHO classification of the gastrointestinal tract (16), the present study defined SRC as cases in which the adenocarcinoma constituted >50% signet ring cells. Li *et al* (6) reported 14 patients presenting with ampullary SRC, including eight males and six females. The median age of the published cases was 57 years (range, 32–83 years) and the median tumor diameter was 1.8 cm (range, 0.8–2.5 cm). The current study reports SRC of the ampulla of Vater, which represents a rare entity accounting for only 4.9% of all ampullary cancers. Furthermore, no sex predilection was observed in the distribution of SRC; males and females were equally affected in the eight cases. The mean patient age at diagnosis was 60 years (range, 42–75 years). Owing to its location at the ampulla, obstructive jaundice was the most common symptom. The majority of patients also exhibited dilation of the common biliary duct and the main pancreatic duct, as well as an enhanced mass lesion in the ampulla of Vater, as determined by helical CT. The tumor size ranged between 1.0 and 9.5 cm (mean size, 3.8 cm).

It is well known that SRC is an extremely rare histological subtype of adenocarcinoma, which is normally found in the gastrointestinal tract, particularly in the stomach. Due to its association with the gastric epithelium, Blundell *et al* (13) hypothesized the origin of ampullary SRC only in the presence of gastric mucosa/metaplasia. Furthermore, Bakkelund *et al* (18) demonstrated that SRCs originate from neuroendocrine cells in gastric cancers. One author has also described a double-secreting amphicrine tumor with a large population of neuroendocrine cells in the ampullary SRC (19). The majority of studies have attempted to discuss the cellular origin and differentiation of ampullary SRC based on the immunohistochemical staining of CK and MUC. de Paiva Haddadd *et al* (20) demonstrated that MUC1 and CK7 were associated with the PB phenotype; whereas the expression of CK20, MUC2 and CDX2 were significantly in ampullary tumors of the I-type than that in PB-type. In addition, the expression of CD10 was significantly higher in I tumors (20). MUC5AC and MUC6 coexpression has been regarded to represent gastric differentiation (21). According to immunohistochemical classification systems, Kawabata *et al* (22) attempted to classify ampullary carcinomas into the following three types: I, PB and unusual types. With regard to the different histological phenotypes, the present study attempted to establish a simple ampullary SRC classification system based on the immunohistochemical staining of CK and MUC. In total, four out of eight cases showed poorly differentiated ampullary adenocarcinomas with prominent signet ring cells floating in the pools of mucous, two of these cases were positive for CK7, CK19 and MUC1, while only one was positive for CK7, CK19, MUC1 and CD10. This was suggestive of SRC arising from the distal section of the ductal pancreatic or biliary epithelium in the former cases (cases 5 and 6) and SRC arising from the I mucosa lining and PB epithelium in the latter (case 3). Additional results indicated that the CK20^+^, CDX2^+^ and MUC2^+^ pattern fully corresponds to the immunohistochemical I type in the one patient with prominent signet ring cells floating in the pool of mucus (case 2). Two of the pure SRCs showed positive expression of CK7, CK19 and MUC1, which indicated that the tumor cells had arisen from PB differentiation (cases 4 and 7); while one case was positive for CK19, MUC2 and MU5AC, which suggested that the pure SRC had arisen from the mixed type, consisting of I muscosa linig and PB epithelium (case 8). In this study, four of the eight ampullary SRC patients were positive for gastric MUC5AC and MUC6, and heterotopic or metaplastic gastric mucosa was observed frequently in the peritumoral lesion in ampullary SRC. These results indicated that specific ampullary SRC may arise from gastric differentiation. According to these results, these tumors were classified into the following four types: I, PB, gastric and mixed types (composed of I and PB epithelium). The gastric/pyloric-type epithelium is frequently observed in intraductal lesions of the pancreas, particularly in intraductal papillary mucinous neoplasm composed of the I and PB epithelium. Chetty and Serra (23) suggested that gastric/pyloric metaplasia of the pancreatic ductal epithelium is a common and perhaps pivotal event in the pathogenesis of intraductal lesions. It also indicated a close pathogenic correlation between the I/PB and gastric phenotypes in ampullary SRC. In this study, all of cases showed no neuroendocrine differentiation.

Hsu *et al* (24) described that the loss of expression of the E-cadherin/β-catenin complex does not correlate with less differentiated histology and poor prognosis in ampullary cancer. Patients whose tissues showed membranous staining of E-cadherin exhibit a long-term result similar to that of aberrant cytoplasmic expression. Park *et al* (25) found that the nuclear accumulation of β-catenin expression correlates with protruding growth and the well-differentiated type. The authors also reported that the membranous loss of β-catenin is associated with poor survival rate. In the gastrointestinal tract, the majority of SRCs exhibit a loss of E-cadherin and β-catenin. However, in the present study, three out of four of the PB-type ampullary SRCs exhibited membranous staining of E-cadherin and β-catenin (cases 4, 5 and 7). This may suggest that E-cadherin and β-catenin coexpression results from the activation of an additional oncogenic pathway that induces carcinogenesis in the PB-type ampullary SRC. The carcinogenesis of ampullary SRC may differ from that of other gastrointestinal malignancies.

In the current study, all cases underwent pancreaticoduodenectomy and only one patient underwent extended lymphadenectomy. The patients with pure SRC showed an improved five-year survival time (mean, 51 months) than that of patients with the mixed-type, poorly differentiated adenocarcinoma (mean, 26 months). The clinical follow-up of I-type ampullary SRC patients revealed a more favorable prognosis than that for patients with PB-type ampullary SRC differentiation. The patients with mixed-type ampullary SRC may have a poorer prognosis than the other phenotypes. Furthermore, the coexpression of E-cadherin and β-catenin revealed a poor prognosis in ampullary SRC.

In conclusion, the current study presents eight cases of SRC in the ampulla of Vater with regard to the detailed clinicopathological features and immunohistochemical phenotypes. In addition, the histological origin and prognosis is discussed and the correlation between the immunohistochemical phenotypes and survival is evaluated. Although the majority of the cases were considered to be the PB type, as determined from the results of the immunohistochemical staining, there is a possible pathogenic correlation between gastric-type ampullary SRC, and MU5AC and MUC6 expression. In contrast to gastric SRC, E-cadherin and β-catenin were positive in ampullary SRC, indicating that the carcinogenesis of ampullary SRC may differ from that of other gastrointestinal malignancies. However, a limitation of this study was the lack of genetic study according to the suggested testing algorithm for identifying the disease category. Furthermore, additional investigation is required to confirm its histological origin and to discuss the correlation between the clinicopathological features and differentiation.

## Figures and Tables

**Table I:** Clinical features of eight patients with signet ring cell carcinoma lesions.

Case	Age, years/gender	Clinical presentation	CT/MRI	Treatment following PD	Follow-up status (months)
1	40/F	Jaundice, intermittent right abdominal pain	Dilated CBD with soft tissue mass at ampulla	ACT	A (8)
2	64/F	Fever, weight loss	Dilated CBD mass in ampullary region	None	A (76)
3	75/F	Jaundice, weight loss, upper abdominal pain	Peri-ampullary mass	ACT/ART	D (16)
4	62/M	Jaundice, fever, abdominal pain, vomiting	Dilated CBD mass in ampullary region	None	D (27)
5	62/M	Fever, vomiting, diarrhea	Dilated CBD with wall thickening	None	D (9)
6	53/M	Asymptomatic	Diffuse ampullary wall thickening	ACT	A (45)
7	66/F	Jaundice, diarrhea	Dilated CBD mass in ampullary region	None	A (54)
8	68/M	Jaundice, weight loss	Peri-ampullary mass	ACT	A (72)

CT, computed tomography; MRI, magnetic resonance imaging; PD, pancreaticoduodenectomy; F, female; M, male; CBD, common biliary duct; ACT, adjuvant chemotherapy; ART, adjuvant radiotherapy; A, alive; D, deceased.

**Table II:** Pathological characteristics of signet ring cell carcinoma lesions.

Case	Tumor size, cm	Lymphatic invasion	Vascular invasion	Histology	Stage^a^
1	3.0×2.0	P	P	Por	IIA
2	6.5×4.2	P	P	Pfm	III
3	3.0×3.5	P	N	Por	III
4	2.4×2.0	P	P	Pur	IIB
5	3.0×2.0	P	N	Por	IIB
6	1.2×0.7	N	N	Por	IIA
7	1.5×1.4	N	N	Pur	IIA
8	9.5×5.5	P	N	Pur	III

a According to the World Health Organization staging system.

P, positive; N, negative; Por, poorly differentiated adenocarcinoma composed of prominent signet ring cells; Pur, pure signet ring; Pfm, prominent signet ring cells floating within the mucin.

**Table III:** Immunohistochemical findings.

Case	CK7	CK19	CK20	CDX2	MUC1	MUC2	MU5AC	MUC6	β-catenin	CD10	E-cad
1	−	+	−	−	−	−	+/−	+	−	−	−
2	−	−	+	+	−	+	−	−	−	−	−
3	+/−	+	−	−	+	−	+/−	+/−	−	+	−
4	+	+	−	−	+	−	+	+	+	−	+
5	+	+	−	−	+	−	+	+	+	−	+
6	+	+	−	−	+/−	−	+	−	−	−	−
7	+	+	−	−	+/−	−	+	−	+	−	+
8	−	+	−	−	−	+	+	−	−	−	−

CK, cytokeratin; CDX2, caudal-related homeobox transcription factor 2; MUC, mucin; E-cad, E-cadherin; −, negative; +, positive; +/−, focal positive.

## References

[1] Howe JR, Klimstra DS, Moccia RD, Conlon KC, Brennan MF (1998). Factors predictive of survival in ampullary carcinoma. Ann Surg.

[2] Albores-Saavedra J, Henson DE, Klimstra DS (2000). Tumors of the gallbladder, extrahepatic bile duct, and ampulla of Vater. Atlas of Tumor Pathology.

[3] Sekoguchi T, Mizumoto R (1979). Clinicopathological study of papilla of Vater. Geka Chiryo.

[4] Eriguchi N, Aoyagi S, Jimi A (2003). Signet ring cell carcinoma of the ampulla of Vater: report of a case. Surg Today.

[5] Ramia JM, Mansilla A, Villar J (2004). Signet-ring-cell carcinoma of the Vater’s ampulla. JOP.

[6] Li L, Chen QH, Sullivan JD (2004). Signet-ring cell carcinoma of the ampulla of Vater. Ann Clin Lab Sci.

[7] Bloomston M, Walker M, Frankel WL (2006). Radical resection in signet ring carcinoma of the ampulla of Vater: report of an 11-year survivor. Am Surg.

[8] Akatsu T, Aiura K, Takahashi S (2007). Signet-ring cell carcinoma of the ampulla of Vater: report of a case. Surg Today.

[9] Gao JM, Tang SS, Fu W, Fan R (2009). Signet-ring cell carcinoma of ampulla of Vater: contrast-enhanced ultrasound findings. World J Gastroenterol.

[10] Maekawa H, Sakurada M, Orita H, Sato K (2011). Signet-ring cell carcinoma co-existing with adenocarcinoma of the ampulla of vater. JOP.

[11] Daoudi K, El Haoudi K, Bouyahia N (2012). Signet ring cell carcinoma of the Vater’s ampulla: A very rare malignancy. Case Rep Oncol Med.

[12] Hara T, Kawashima H, Ishigooka M (2002). Signet-ring-cell carcinoma of the ampulla of Vater: a case report. Hepatogastroenterology.

[13] Blundell CR, Kanun CS, Earnest DL (1982). Biliary obstruction by heterotopic gastric mucosa at the ampulla of Vater. Am J Gastroenterol.

[14] Zhou H, Schaefer N, Wolff M, Fischer HP (2004). Carcinoma of the ampullary of Vater: comparative histologic/immunohistochemical classification and follow-up. Am J Surg Pathol.

[15] Gheza F, Cervi E, Pulcini G (2011). Signet ring cell carcinoma of the ampulla of Vater: demonstration of a pancreatobiliary origin. Pancreas.

[16] Hamilton SR, Nakamura S, Bosman FT, Quirke P, Boffetta P, Riboli E, IIyas M, Sobin LH, Morreau H, Bosman FT, Carneiro F, Hruban RH, Theise ND (2010). Carcinoma of the colon and rectum. WHO Classifcation of Tumours of the Digestive Organs.

[17] Yokota T, Kunii Y, Teshima S (1998). Signet ring cell carcinoma of the stomach: a clinicopathological comparison with the other histological types. Tohoku J Exp Med.

[18] Bakkelund K, Fossmark R, Nordrum I, Waldum H (2006). Signet ring cells in gastric carcinomas are derived from neuroendocrine cells. J Histochem Cytochem.

[19] Gardner HA, Matthews J, Ciano PS (1990). A signet-ring cell carcinoma of the ampulla of Vater. Arch Pathol Lab Med.

[20] de Paiva Haddad LB, Patzina RA, Penteado S (2010). Lymph node involvement and not the histophatologic subtype is correlated with outcome after resection of adenocarcinoma of the ampulla of vater. J Gastrointest Surg.

[21] Gürbüz Y, Klöppel G (2004). Differentiation pathways in duodenal and ampullary carcinoma: a comparative study on mucin and trefoil peptide expression, including gastric and colon carcinomas. Virchow Arch.

[22] Kawabata Y, Tanaka T, Nishisaka T (2010). Cytokeratin 20 (CK20) and apomucin 1 (MUC1) expression in ampullary carcinoma: Correlation with tumor progression and prognosis. Diagn Pathol.

[23] Chetty R, Serra S (2009). Intraductal tubular adenoma (pyloric gland-type) of the pancreas: a reappraisal and possible relationship with gastric-type intraductal papillary mucinous neoplasm. Histopathology.

[24] Hsu HP, Shan YS, Jin YT (2010). Loss of E-cadherin and beta-catenin is correlated with poor prognosis of ampullary neoplasms. J Surg Oncol.

[25] Park S, Kim SW, Lee BL (2006). Expression of E-cadherin and beta-catenin in the adenoma-carcinoma sequence of ampulla of Vater cancer. Hepatogastroenterology.

